# Scalp hair cortisol and testosterone levels in patients with sarcoidosis

**DOI:** 10.1371/journal.pone.0215763

**Published:** 2019-06-14

**Authors:** M. J. G. van Manen, V. L. Wester, E. F. C. van Rossum, L. M. van den Toorn, K. Y. Dorst, Y. B. de Rijke, M. S. Wijsenbeek

**Affiliations:** 1 Department of Respiratory Medicine, Erasmus MC, University Medical Center, Rotterdam, the Netherlands; 2 Department of Internal Medicine, Erasmus MC, University Medical Center, Rotterdam, the Netherlands; 3 Department of Clinical Chemistry, Erasmus MC, University Medical Center, Rotterdam, the Netherlands; Chiba Daigaku, JAPAN

## Abstract

**Background:**

Patients with sarcoidosis often experience fatigue and psychological distress, but little is known about the etiology of these conditions. While serum and saliva steroid hormones are used to monitor acute steroid levels, scalp hair analysis is a relatively new method enabling measurement of long-term steroid levels, including hair cortisol reflecting chronic stress. We investigated whether scalp hair cortisol and testosterone levels differ between sarcoidosis patients both with and without fatigue and general population controls. Additionally, we studied if these hormones could serve as objective biomarkers for psychological distress in patients with sarcoidosis.

**Methods:**

We measured hair steroid levels using liquid chromatography–tandem mass spectrometry in glucocorticoid naïve sarcoidosis patients. Patients completed the Perceived Stress Scale, Fatigue Assessment Scale, Hospital Anxiety and Depression Scale and Short Form 36 (SF-36). Hair steroid levels from 293 participants of the population-based Lifelines cohort study served as controls.

**Results:**

Thirty-two patients (14 males) were included. Hair cortisol, but not testosterone, concentrations were significantly higher in patients with sarcoidosis than in general population controls (mean 6.6 versus 2.7 pg/mg, p<0.001). No differences were found in hair cortisol and testosterone levels between fatigued and non-fatigued patients with sarcoidosis. Hair cortisol of sarcoidosis patients correlated significantly with anxiety (r = 0.47, p = 0.01), depression (r = 0.46, p = 0.01), and SF-36 mental domain (r = -0.38, p = 0.03), but not with fatigue.

**Conclusions:**

Patients with sarcoidosis have chronically higher levels of the stress hormone cortisol than the normal population, while testosterone levels in hair did not differ. Hair cortisol levels were positively related to subjective measures of psychological distress, but not to fatigue. Our study shows that hair cortisol is a promising non-invasive biomarker for psychological distress in patients with sarcoidosis.

**Trial registration:**

ClinicalTrials.gov: NCT03108547. Registered 31 March 2017, retrospectively registered.

## Introduction

Sarcoidosis is a multi-organ disease of unknown etiology, characterized by non-caseating granulomas, which commonly occur in the lymph nodes, lungs, skin and eyes [1]. In patients with sarcoidosis, fatigue and psychological distress are frequently reported problems [2–5]. Fatigue has been reported in 50–70% of the sarcoidosis patients [3]. Even when sarcoidosis is in clinical remission, fatigue may persist chronically and cause impaired quality of life and reduced socio-economical participation [6]. The exact mechanism for chronic fatigue in sarcoidosis is currently unknown.

Stress might be one of the factors contributing to the onset of fatigue. Chronic fatigue in sarcoidosis patients has been associated with increased levels of perceived stress and psychological stressors such as depression and anxiety [4–6]. Psychological and physical stressors lead to activation of the hypothalamus-pituitary-adrenal axis, resulting in an increase in circulating levels of the stress hormone cortisol [7]. Cortisol, in turn, influences a wide range of bodily functions including metabolism, behavior and immunity [7], and increased cortisol levels can also lead to psychological disturbances, such as anxiety and depressive symptoms. Stress can be assessed subjectively using validated questionnaires. In addition, cortisol levels can be used as a biomarker for stress [8–10]. Systemic inflammation in chronic diseases has been associated with fatigue and may affect cortisol levels as well [11, 12]. While stress and inflammation are both associated with increased levels of cortisol, there are also studies that show evidence of hypocortisolism in relation to chronic fatigue, [13, 14] thereby leaving the relationship between cortisol and fatigue in sarcoidosis unclear.

In male patients with sarcoidosis, another potential factor contributing to fatigue may be hypogonadism, with its consequent low levels of testosterone. Serum testosterone has shown to be significantly decreased in patients with chronic lung disease, [15, 16] and has also been inversely related to fatigue in patients with advanced cancer and obstructive sleep apnea [16, 17]. In a study of 30 patients with sarcoidosis, almost half of the patients had lower serum testosterone levels than healthy controls; however, this study did not assess the relationship with fatigue [18].

Both cortisol and testosterone are commonly measured in blood, urine, or saliva. However, these tests reflect only short-term exposure, and levels of cortisol and testosterone can greatly fluctuate within and across days [7, 19]. Moreover, the tests themselves can induce stress and increase cortisol levels [20].

In this study, we use the relatively novel method of scalp hair analysis to measure long-term cortisol, cortisone, and testosterone levels. This method has been validated in other—non-pulmonary—diseases and allows a retrospective measurement of the endogenous production of these hormones over months of time, based on an average hair growth of approximately 1 cm per month [21]. Previous studies have shown that hair cortisol can serve as a biomarker for chronic stress [9, 10].

To the best of our knowledge, no studies have investigated hair steroid biomarkers, such as cortisol, cortisone and testosterone, in sarcoidosis. This non-invasive method could potentially give new insights into the mechanism of fatigue and psychological distress in sarcoidosis. Moreover, it may be used as a screening and follow-up tool to objectively measure fatigue and psychological distress as an alternative to the currently used subjective patients-reported outcome measures.

In this explorative study, we aimed to investigate whether scalp hair cortisol, cortisone, and testosterone levels differ between sarcoidosis patients both with and without fatigue and general population controls. Additionally, we studied if these scalp hair steroid levels could serve as an objective biomarker for psychological distress, fatigue and/or other clinical outcomes in patients with sarcoidosis.

## Materials and methods

### Study design and population

We conducted a prospective observational study. Sarcoidosis patients were recruited during their regular follow ups at the outpatient clinic of the pulmonary department of the Erasmus University Medical Center, Rotterdam, the Netherlands, from June till December 2014. Patients aged 18 or older were included if they had been diagnosed with sarcoidosis according to the latest ATS/ERS/WASOG statement on sarcoidosis, [1] and if they had sufficient knowledge of the Dutch language. We aimed to include 20 patients with fatigue and 10 without fatigue. Fatigue was defined as a score of 22 or higher on the Fatigue Assessment Scale (FAS) [22]. Other causes of fatigue had to be excluded or, in the case of contributing comorbidities (e.g. obstructive sleep apnea syndrome, hypothyroidism or anemia), optimally treated. Patients were excluded if they had a hair length of less than 1 cm, had used systemic and/or inhalation steroids in the last year, or had taken methylphenidate less than 1 month before the study. Scalp hair steroid levels of participants of the Dutch population-based Lifelines cohort study were used as general population controls [23, 24]. Formal consultation with the Medical Ethical Committee of the Erasmus Medical Center affirmed that, under the Dutch act for medical research involving human subjects (Wet Medisch Onderzoek), approval of this study by the Medical Ethical Committee was not required (MEC-2014-206). All patients gave written informed consent.

The datasets of the sarcoidosis population and general population controls as well as the Pearson Correlation data of Fig 4 are available as supplementary materials.

### Procedures

#### Hair sample collection and processing

**Fig 1:**
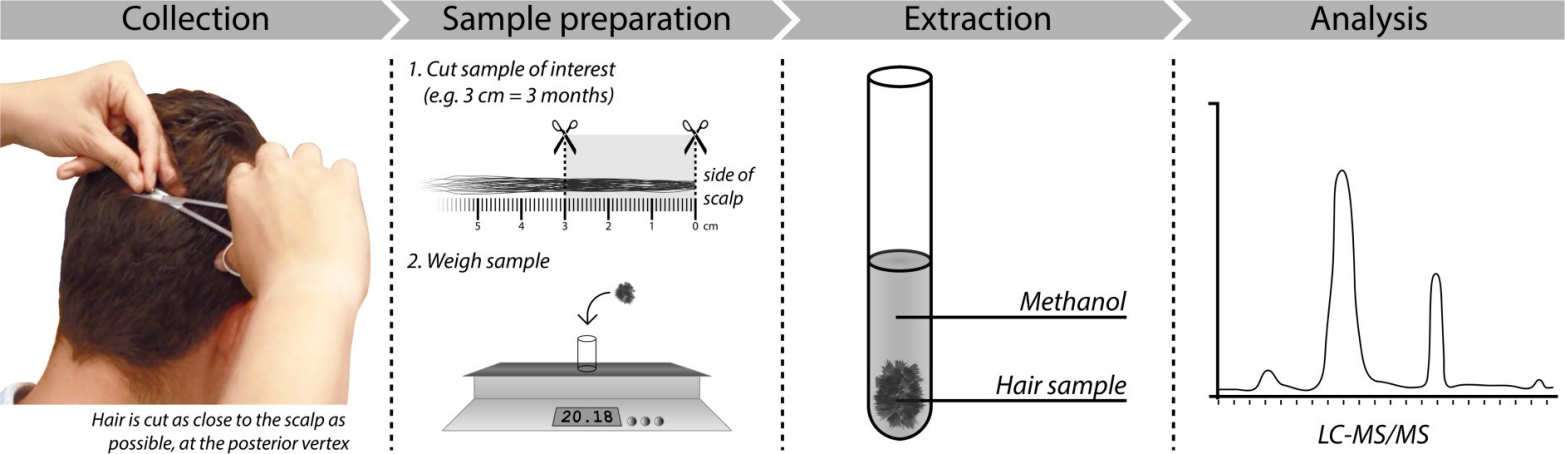
Scalp hair analysis: Hair sample collection, pre-treatment and analysis. LC-MS/MS, liquid chromatography-tandem mass spectrometry. Modified from [21] with permission from the publisher.

In each subject, a small hair sample of approximately 150 hairs was cut from the posterior vertex as close to the scalp as possible using small scissors. Hair samples were taped on paper and stored in envelopes at room temperature. The hair samples of all patients were analyzed in one batch at the department of clinical chemistry of the Erasmus MC, Rotterdam. For analyses, hair samples were divided in segments of 1 cm, weighed, washed, and steroids were extracted with methanol. Subsequently, hair cortisol, cortisone, and testosterone were analyzed with liquid chromatography-tandem mass spectrometry (LC-MS/MS) (Fig 1). A detailed description on scalp hair analysis is available in a previous publication by Noppe and de Rijke et al [25].

#### Questionnaires

Patients filled in the FAS at the outpatient clinic. Other questionnaires were completed at home as soon as possible, but within a maximum of two weeks from the clinic visit. The 10-item FAS measures fatigue and has been well-validated in patients with sarcoidosis [3, 26]. Scores range from 10 to 50, with higher scores indicating more fatigue, a FAS score of ≥ 22 is considered as significant fatigue [27]. Subjective health status was evaluated with standardized self-reported generic instruments, the Euroqol-5D 5-level (EQ5D5L), and the 36-item medical outcomes short form 36 (SF-36), with higher scores representing better health status [28]. The Kings’ Sarcoidosis Questionnaire (KSQ) was used to measure disease-specific health status and consists of five modules: General health status, Lung, Medication, Skin, and Eye [29, 30]. All domain scores range from 0 to 100, with a higher score representing a better disease-specific health status [29]. The 14-item Perceived Stress Scale (PSS) is a broad score to assess a person’s perception of stress, with a higher score indicating a higher perceived stress [8]. The Hospital Anxiety and Depression Scale (HADS) has a 7-item anxiety and a 7-item depression domain. The scores for both domains range from 0–21, with a cut-off point of 8 for depression or anxiety [31]. Patients also completed questions regarding artificial hair coloring and frequency of hair washing.

#### Clinical parameters

Weight, height and waist circumference were measured during the visit. The results of routinely measured pulmonary function outcomes (forced vital capacity (FVC) and transfer factor for carbon monoxide corrected for hemoglobin (TLCOc)) were used when available.

#### Disease activity

Two ILD doctors independently rated disease activity of sarcoidosis. Criteria for determining disease activity were based on literature search and ILD expert opinions. A patient was considered having active disease when at least one of the following criteria was fulfilled: 1. Progressive loss of organ function due to sarcoidosis (such as pulmonary function, liver and kidney function, serum calcium concentration, eye involvement, hart function etc.); 2. Evidence of disease activity on PET-scan or SMS scans and/or increased serum ACE or sIL2R; 3. Clinical signs of disease activity during physical examination (such as fever, erythema nodosum, uveitis anterior, lymphadenopathy etc.).

#### Outcomes

The primary outcomes are the differences between scalp hair steroid levels of sarcoidosis patients both with and without fatigue and general population controls. Secondary outcomes are relationships between scalp hair steroid levels, fatigue and psychological distress scores, and clinical parameters.

### Statistical analysis

Data are presented as median (range) when not stated differently. Scalp hair steroid levels are presented as geometric mean (95% confidence interval, CI) and expressed in pg/mg; data were log-transformed to achieve normal distribution. To determine differences in characteristics of sarcoidosis patients with and without fatigue, we used a Mann-Whitney U test for continuous variables and Fisher’s exact test for binomial variables. Hair steroid levels were compared between the two groups of sarcoidosis patients, and between sarcoidosis patients with and without active disease using independent sample t-tests. Analysis of (co)variance (AN[C]OVA) was used to compare hair cortisol and testosterone levels of sarcoidosis patients with general population controls, corrected for age and gender. Mann Whitney U tests were used for analyzing differences in questionnaire scores of patients with and without fatigue. Correlations between hair steroid levels, questionnaire scores and clinical parameters were assessed using Pearson’s correlation coefficients. All statistical analyses were performed using SPSS version 21.0 (IBM, Armonk, NY). A p-value of <0.05 was considered statistically significant.

## Results

Of the 36 patients screened for the study, 32 were included. Patients were excluded for the following reasons: incomplete questionnaires (2), not enough hair for hair sample (1), and known psychiatric problems (1). Based on the FAS score, 23 of the 32 sarcoidosis patients had significant fatigue. There were no significant differences in patients’ characteristics between the fatigued and non-fatigued groups of patients (Table 1).

**Table 1:** Characteristics of the groups of sarcoidosis patients.

	All patients [n = 32]	Fatigue [n = 23]	Non-fatigue [n = 9]	p-value*
Fatigue severity	32 (10–46)	33 (22–46)	16 (10–21)	<0.001
Age	47 (31–66)	45 (31–64)	55 (36–66)	0.15
Women	18 (56)	13 (57)	5 (56)	1.00
Ethnicity				0.69^#^
Caucasian	23 (72)	17 (74)	6 (67)	
Moroccan	3 (9)	3 (13)	0 (0)	
Surinamese Hindi	6 (19)	3 (13)	3 (33)	
BMI, kg/m^2^	26 (20–39)	26 (20–39)	29 (23–36)	0.21
Waist circumference	93 (65–116)	91 (65–116)	95 (84–104)	0.48
Time since diagnosis, y	5 (0–24)	4 (0–24)	6 (0–18)	0.82
FVC % predicted	105 (55–123)	103 (81–123)	106 (55–121)	0.95
TLCOc % predicted	88 (50–162)	89 (50–131)	86 (60–162)	0.82
Active Disease	24 (75)	17 (74)	7 (78)	1.00

Data are presented as median (range) or n (%). BMI, body mass index; FVC, forced vital capacity; TLCOc transfer factor for carbon monoxide corrected for haemoglobin

* Mann-Whitney U test was used for continuous variables and Fisher’s exact test for binomial variables to compare fatigue and non-fatigue sarcoidosis patients

^#^ Caucasian versus other

### Hair steroid levels

**Fig 2:**
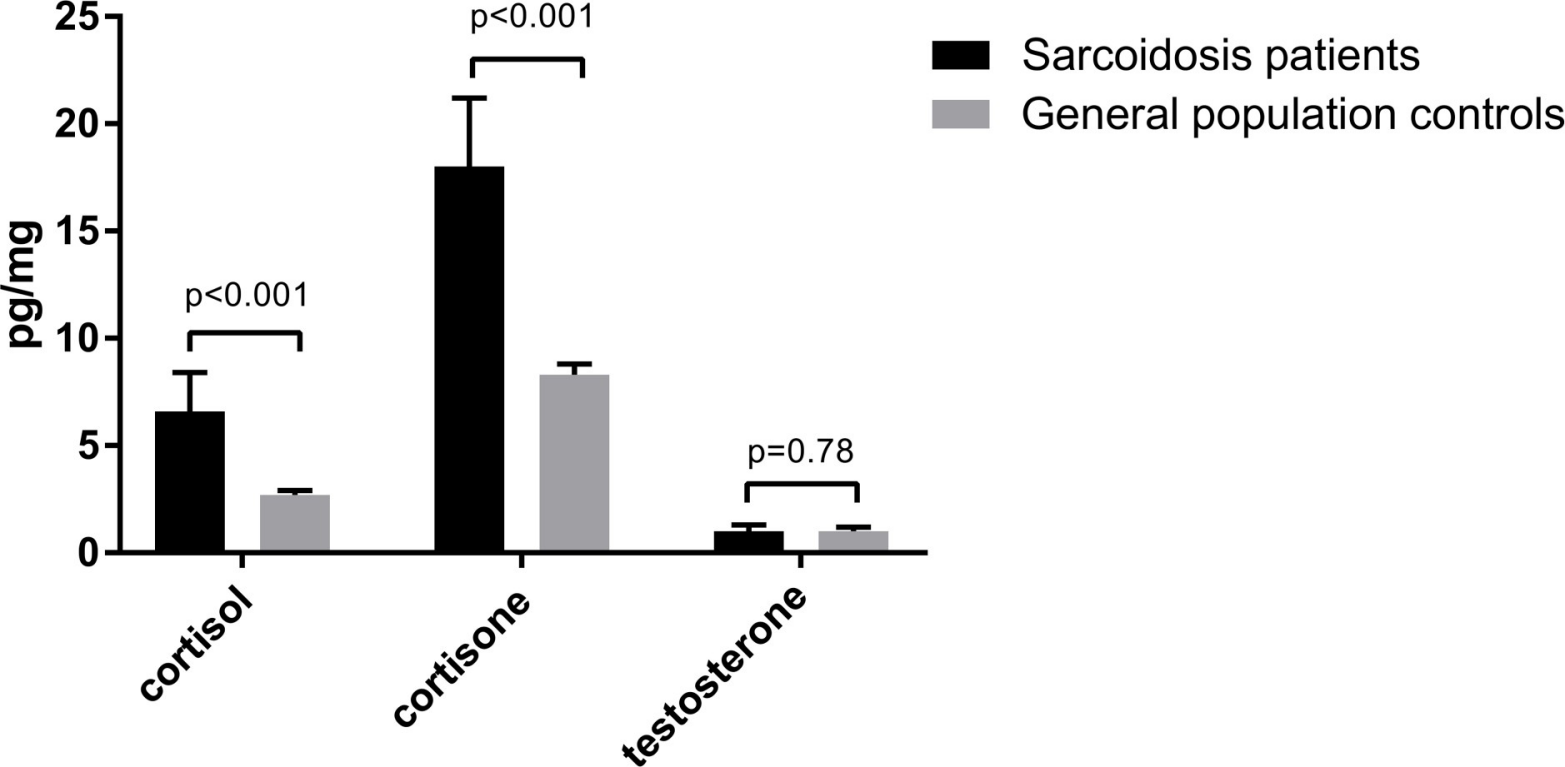
Hair cortisol, cortisone and testosterone levels in patients with sarcoidosis and general population controls [23, 24]. Cortisol and cortisone were measured in 32 sarcoidosis patients and in 293 general population controls. Testosterone was measured in 13 male sarcoidosis patients and in 62 male general population controls. Data are shown as geometric mean (95% CI), and corrected for age and gender. General Linear model–Univariate (ANCOVA) was used for analysis.

**Fig 3:**
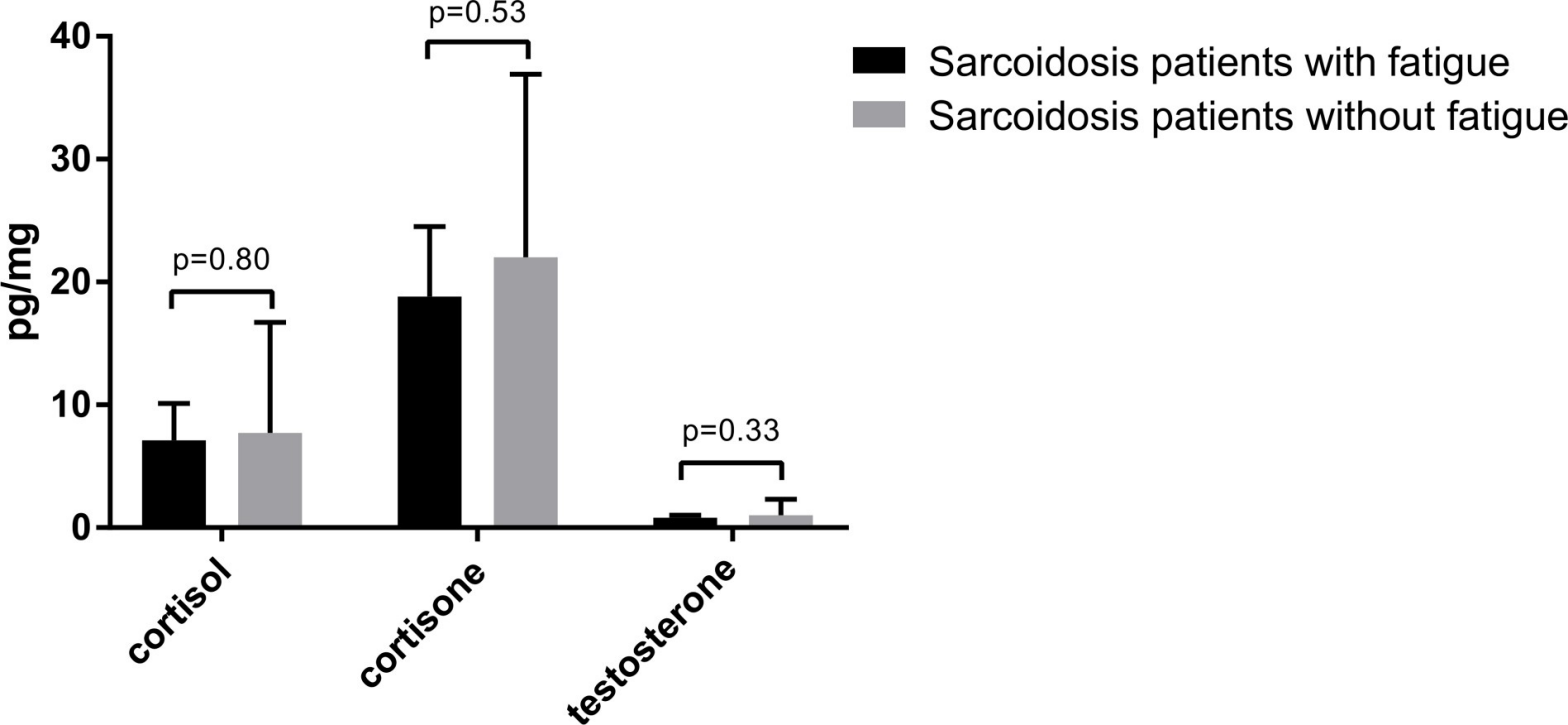
Hair cortisol, cortisone and testosterone levels in sarcoidosis patients with and without fatigue. Cortisol and cortisone were measured in 23 sarcoidosis patients with fatigue and in 9 patients without fatigue. Testosterone was measured in 10 male sarcoidosis patients with fatigue and in 4 male patients without fatigue. Data are shown as geometric mean (95% CI). Independent sample t-test was used for analysis.

Scalp hair cortisol and cortisone levels were detected in the total of 32 sarcoidosis patients, and testosterone levels in 13 of the 14 male patients with sarcoidosis. In the population-based controls from the Lifelines study cohort, there were 293 participants with scalp hair steroid samples (262 had both cortisol and cortisone levels available, 4 only cortisol level, and 27 only cortisone). Scalp hair cortisol and cortisone concentrations of the total group of sarcoidosis patients were significantly higher than those of the general population controls (hair cortisol: mean 6.6 (95% CI 5.1–8.4) versus 2.7 (95% CI 2.5–2.9) pg/mg, p<0.001, hair cortisone: mean 18.0 (95% CI 15.2–21.2) versus 8.3 (95% CI 7.9–8.8) pg/mg, p<0.001) (Fig 2). In 62 male participants, testosterone levels were also available. No significant differences in testosterone levels were found between male patients with sarcoidosis and male general population controls (mean 1.0 (95% CI 0.7–1.3) pg/mg versus 1.0 (95% CI 0.9–1.2) pg/mg, p = 0.78) (Fig 2). Hair steroid levels in the sarcoidosis patients with fatigue did not significantly differ from those of sarcoidosis patients without fatigue (Fig 3). Cortisol correlated significantly with cortisone (r = 0.72, p<0.001), but not with testosterone (r = 0.38, p = 0.20); neither did cortisone correlate with testosterone (r = 0.26, p = 0.39). Artificial hair coloring and hair washing frequency had no influence on hair steroid levels in patients with sarcoidosis (data not shown).

### Psychological distress and fatigue questionnaires

Mean questionnaires scores are shown in Table 2. Sarcoidosis patients with fatigue had significantly higher scores with respect to stress, anxiety, depression and lower quality of life scores than sarcoidosis patients without fatigue (Table 2).

**Table 2:** Questionnaire scores of patients with sarcoidosis.

Questionnaire	Total [n = 32]	Fatigue [n = 23]	Non-fatigue [n = 9]	p-value*
FAS	32 (10–46)	33 (22–46)	16 (10–21)	<0.001
PSS	26 (4–55)	29 (4–55)	14 (9–28)	<0.001
HADS anxiety	6 (0–19)	7 (0–19)	1 (0–2)	<0.001
HADS depression	6 (0–21)	8 (0–21)	0 (0–5)	<0.001
EQ5D5L	0.7 (-0.1–1.0)	0.7 (-0.1–0.9)	1.0 (0.6–1.0)	<0.001
SF-36 mental health	64 (36–75)	64 (36–75)	64 (60–64)	0.71
SF-36 general health	62 (30–87)	62 (47–87)	57 (30–77)	0.26
KSQ GHS	58 (2–100)	53 (2–83)	98 (75–100)	<0.001

Data are shown as median (range).

*Mann-Whitney U was used for comparing questionnaire scores in fatigue and non-fatigue patients with sarcoidosis. FAS, Fatigue Assessment Scale; PSS, Perceived Stress Scale; HADS, Hospital Anxiety and Depression Scale; EQ5D5L, Euroqol-5D 5-level; SF-36, Short Form-36; KSQ, King's Sarcoidosis Questionnaire; GHS, General Health Status domain

**Fig 4:**
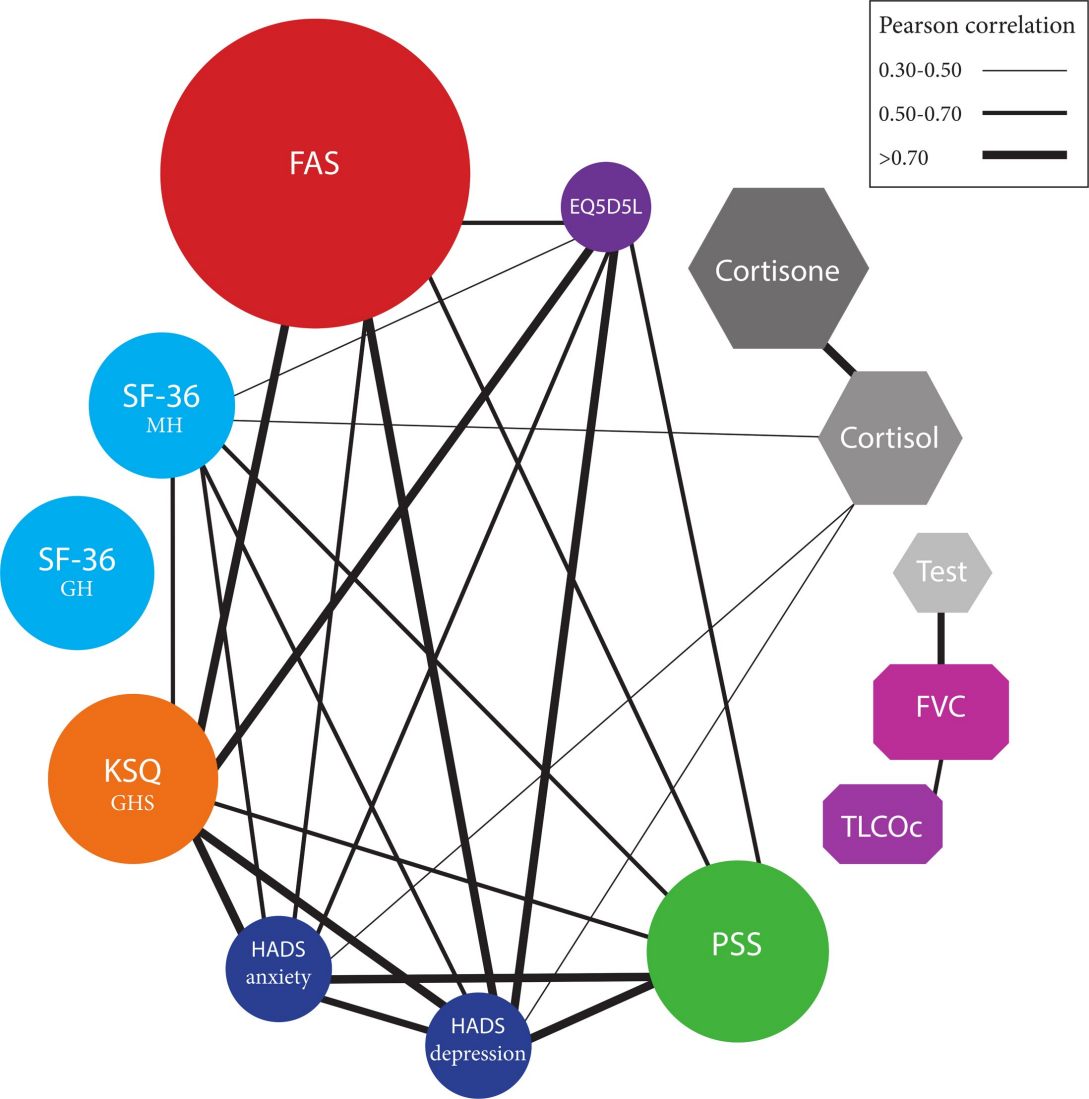
Interaction network showing all significant correlations between hair steroid levels, psychological distress and fatigue scores, and pulmonary function tests in sarcoidosis patients (n = 32). Each node of the network corresponds to a questionnaire score, and its size is proportional to the % of the total score of the questionnaire. Two nodes are linked if they significantly correlate, the wider the string the better the correlation. EQ5D5L; Euroqol-5D 5-level; FAS, Fatigue Assessment Scale; SF-36, Short Form-36; MH, mental health domain; GH, general health domain; KSQ; King's Sarcoidosis Questionnaire; GHS, General Health Status domain; HADS, Hospital Anxiety and Depression Scale; PSS, Perceived Stress Scale; TLCOc, transfer factor for carbon monoxide corrected for hemoglobin; FVC, forced vital capacity; Test, testosterone.

A significantly positive correlation was found between scalp hair cortisol levels and anxiety (r = 0.47, p = 0.01) and depressive symptoms (r = 0.46, p = 0.01) (Table 3). Additionally, higher hair cortisol levels significantly correlated with lower mental health scores, as measured by the SF-36 (r = -0.38, p = 0.03), indicating worse mental health status (Table 3). There was a trend in correlation observed between hair cortisol levels and the PSS (r = 0.31, p = 0.09), but no correlation existed with the FAS (r = 0.14, p = 0.45) (Table 3). For scalp hair testosterone levels, in male sarcoidosis patients, no significant correlations were found with any of the psychological distress and fatigue scores. Fig 4 shows an interaction network between hair steroid levels, psychological distress and fatigue scores, and pulmonary function tests in the total group of sarcoidosis patients.

**Table 3:** Correlations between scalp hair cortisol and cortisone and psychological distress and fatigue scores in all patients with sarcoidosis (n = 32).

Questionnaires	Cortisol	Cortisone
FAS	0.14	-0.01
PSS	0.31^#^	<-0.01
HADS anxiety	0.47**	0.14
HADS depression	0.46**	0.18
EQ5D5L	-0.26	0.03
SF-36 mental health	-0.38*	-0.05
SF-36 general health	-0.28	-0.18
KSQ GHS	-0.25	-0.04

Pearson correlations, data are presented as R (p-value). FAS, Fatigue Assessment Scale; PSS, Perceived Stress Scale; HADS, Hospital Anxiety and Depression Scale; EQ5D5L, Euroqol-5D 5-level; SF-36, Short Form-36, KSQ; King's Sarcoidosis Questionnaire; GHS, General Health Status domain

^#^ p≥0.05 and <0.10

* p<0.05 and >0.01

** p≤0.01

Scalp hair cortisol and cortisone did not correlate with the studied clinical parameters (weight, height, waist circumference and lung function outcomes). However, testosterone showed a significant positive correlation with weight (r = 0.69, p = 0.01), and a significant inverse correlation with waist circumference (r = -0.69, p = 0.01) and FVC (r = -0.76, p = 0.02).

### Disease activity

**Fig 5:**
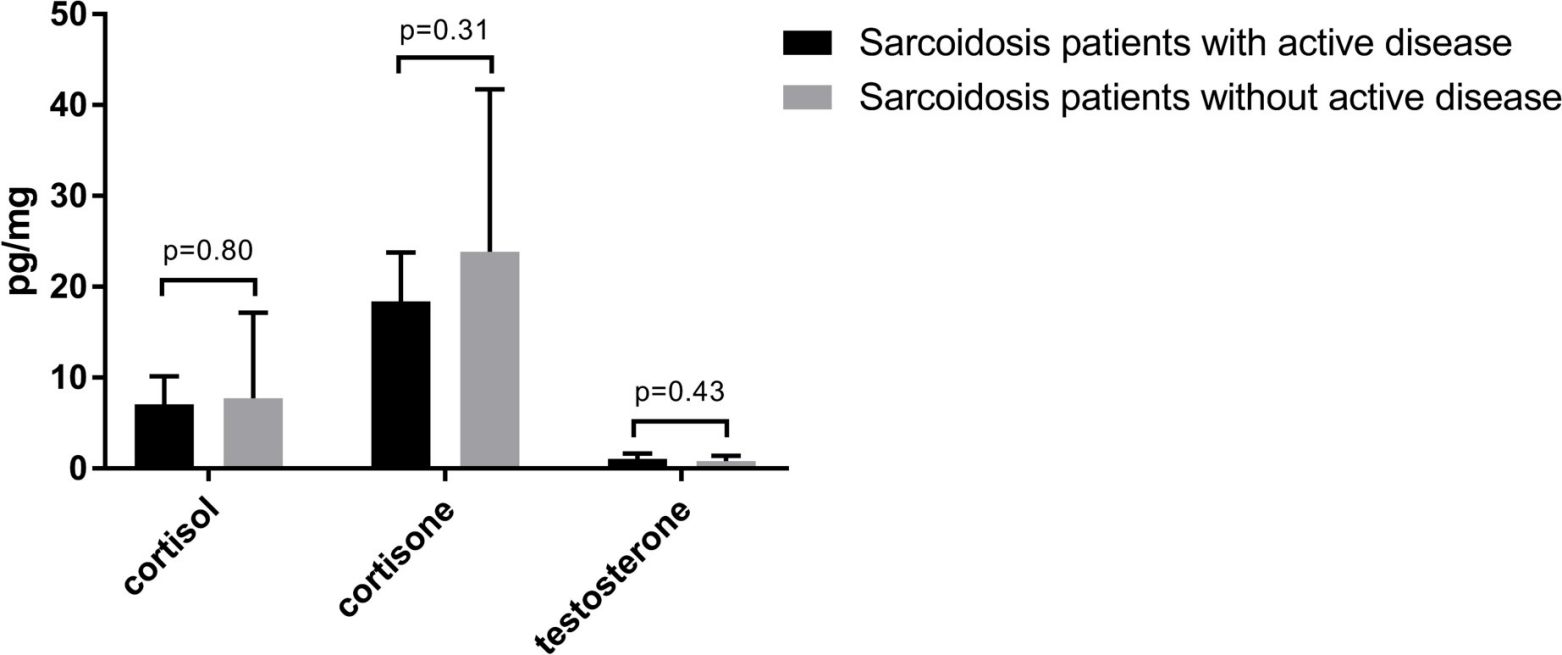
Hair cortisol, cortisone and testosterone levels in sarcoidosis patients with and without active disease. Cortisol and cortisone were measured in 24 sarcoidosis patients with active disease and in 8 patients without active disease. Testosterone was measured in 9 male sarcoidosis patients with active disease and in 4 male patients without active disease. Data are shown as geometric mean (95% CI). Independent sample t-test was used for analysis.

Twenty-four out of 32 patients were rated as having active disease. Agreement between the two independent assessors of disease activity was high, only in three case they had an initial different view. After discussion agreement on disease activity was reached also for these three patients. There were no significant differences in hair cortisol, cortison and testosterone concentrations between patients with and without active disease (Fig 5).

## Discussion

This study is the first to assess scalp hair steroid levels in patients with sarcoidosis. Interestingly, both hair cortisol and cortisone levels were significantly higher in sarcoidosis patients than in general population controls. Hair cortisol levels were positively related to subjective measures of psychological distress. No differences were found in scalp hair steroid levels between sarcoidosis patients with and without fatigue, nor did we observe differences in testosterone levels between patients with or without fatigue and controls.

In the current study, rather than the more conventional methods such as serum, urine or saliva measurements, we used scalp hair analysis to determine steroid levels. This method has several advantages. First, scalp hair analysis enables long-term steroid level determination as opposed to all methods using body fluids that only represent a momentarily steroid level and which can be influenced by acute stress, the diurnal rhythm, or pulsatile secretion of steroid hormones [7, 19, 21]. A recent study has shown that hair cortisol measurement provides a reliable measure of long-term integrated free cortisol production. It demonstrated that hair cortisol levels correlated well with 30-day average salivary cortisol area-under-the curve based on 3 samples collected during the day [32]. Second, scalp hair samples are easy to collect and store at room temperature [33]. In contrast, measuring steroid levels in body fluids, especially at multiple time-points, is invasive for patients, labour-intensive, and depends on patient instruction and cooperation [21]. Also, the measurement itself, as with blood sampling, can induce stress and thereby influence results [20].

Interestingly, hair cortisol and cortisone levels were significantly higher in patients with sarcoidosis than in the general population controls. Hair cortisol has previously been associated with both physical and mental health status and was found to be increased in patients with various diseases such as obesity, post-traumatic stress syndrome, and cardiovascular diseases and was also associated with extreme exercise, shift work, life events and chronic pain [10, 21, 34, 35]. Cortisol is the active form of glucocorticoid and can be converted into the biologically inactive cortisone. In our study, cortisol and cortisone showed good correlations and were both increased. This makes a mechanistic cause in the conversion or balance between cortisol and cortisone in sarcoidosis patients unlikely as the underlying mechanism for the higher cortisol levels found.

Scalp hair steroid levels did not differ between sarcoidosis patients with and without fatigue. Korenromp et al. reported, in a principal component analyses serum, that ACTH and cortisol were significantly lower in patients with sarcoidosis-related fatigue than in patients without fatigue [13]. However, their baseline cortisol analysis showed no difference between fatigued and non-fatigued patients, which is in line with our findings. As Korenromp measured serum cortisol, which is a momentary and highly variable measure, this may well account for the differences found [13]. Previous research already showed that serum cortisol levels do not seem to correlate well with scalp hair cortisol levels [36].

Hair testosterone levels in men with sarcoidosis did not differ from those of the general population controls. This is contrary to findings of Spruit et al. which showed significantly lower serum testosterone levels in male sarcoidosis patients than in healthy controls [18]. This variance could again be due to differences in measuring methods. However, the small sample size (13 testosterone samples) of our study might have also played a role in the findings. Nevertheless, both our study and the study of Spruit et al. found no correlation between psychological wellbeing scores and testosterone levels. We can therefore only conclude that the clinical relevance of testosterone levels in sarcoidosis is not yet clear, and ideally a larger cohort should be studied.

Interestingly, in our study, scalp hair cortisol levels positively correlated with psychological distress scores of depression and anxiety. A trend was found in the correlation with perceived stress scores. These findings are in line with previous studies [33, 37]. The underlying mechanism of the positive correlation between scalp hair cortisol levels and psychological distress in sarcoidosis patients could theoretically be bi-directional. Sarcoidosis-associated inflammation could cause increased cortisol levels, and increased cortisol levels can lead to psychological distress. Studies in both Cushing’s patients (with severe endogenous hypocortisolism) or users of high dose exogenous corticosteroids clearly show that elevated corticosteroid exposure can lead to anxiety, depressive symptoms and other forms of psychological distress [38, 39]. However, the data of our study are not in support of this theory; no differences in cortisol levels were found in patients with and without disease activity. An alternative explanation could be that the burden of having such a disease as sarcoidosis directly causes psychological distress, resulting in higher cortisol levels. Our results are in line with this hypothesis, since symptoms of anxiety and depression were positively associated with cortisol levels in sarcoidosis patients. In fatigued and non-fatigued patients, no differences in cortisol levels were found. This might indicate that fatigue is not the predominant underlying cause of the increase in cortisol levels found in sarcoidosis patients. Other factors which we did not assess extensively in our study such as, sleep, chronic pain (joint involvement of the disease) or dietary pattern may also contribute to the increased cortisol levels we observed in sarcoidosis patients.

The relationship found between hair cortisol levels and psychological distress may have clinical implications. Interventions aimed at improving quality of life for patients with sarcoidosis and other lung diseases have shown to have a beneficial effect on quality of life, but little is known about their effect on psychological stress [40–43]. It shows the need for future studies with more emphasis on psychological distress that patients with sarcoidosis experience. Furthermore, scalp hair cortisol may be used in future as an objective long-term biomarker for chronic stress which could complement or even replace subjective patient-reported outcome measures (PROMs). PROMs often comprise many questions, which may complicate use in clinical practice. Not only can completing PROMS be time consuming, but patients may also struggle to remember what they answered the previous time. This can lead to variations in scores and difficulties to evaluate a meaningful change. Previous studies have shown that scalp hair cortisol can serve as a biological marker for chronic stress. Scalp hair can also be divided into segments of one or more centimetres to create retrospective timelines. In this way, scalp hair steroids could potentially be used in future trials to objectively measure the effect of interventions on psychological distress using only one or a few hair strings, thereby allowing easy data collection on a large scale.

Our results, together with those from others, underline again how little we unfortunately still understand about the etiology of fatigue in sarcoidosis. Fatigue is presumably multifactorial, and might be related to aspects in pathogenesis, comorbidities, and medication [2, 3]. In the current study, no relationship could be found between cortisol and testosterone levels and fatigue. This is in line with clinical experience that, in sarcoidosis-associated fatigue, steroids treatment is often futile, or might even result in side-effects causing further deterioration. As can been seen in Fig 4, fatigue is closely related to quality of life, anxiety, depression and stress scores, which, in turn, are also strongly interrelated. This stresses the major impact fatigue has on patients' lives and the need for studies to advance knowledge on its etiology and for effective treatment interventions for fatigue in sarcoidosis.

Fig 4 also shows that scalp hair cortisol is the only objective clinical parameter correlating with subjective scores of psychological distress. Importantly, no correlation is found between questionnaires and pulmonary function parameters. This is in line with previous findings that pulmonary function tests do not correlate with subjective questionnaire scores as they capture another dimension of disease [29, 30]. Testosterone levels showed a correlation with FVC in sarcoidosis patients; however, sample size was limited and too small to draw conclusions about the clinical significance of this finding.

Our study has some limitations. Only patients without steroid use were included, whereas in daily practice, both systemic and topical corticosteroids are used by a fair number of patients with sarcoidosis. Previous studies have shown that endogenous cortisol production can be suppressed by exogenous corticosteroids [44]. In healthy adults, significantly lower hair cortisol levels were found in participants using systemic or local corticosteroids than in non-users [24, 33]. It needs to be studied if scalp hair cortisol analysis can be used to assess compliance and dosing of corticosteroid treatment in sarcoidosis. Furthermore, as no official criteria exist for disease activity in sarcoidosis, we used a score based on expert opinion and literature. Another limitation is the small sample size, which might explain the lack of differences found in hair steroid levels in fatigued and non-fatigued patients. However, in our study we showed that hair steroid levels of the total group of patients differed significantly from those of the general population controls.

## Conclusions

Patients with sarcoidosis have significantly higher levels of scalp hair cortisol and cortisone than the general population controls, while testosterone levels in hair did not differ from these controls. Scalp hair steroids levels did not differ in sarcoidosis patients with and without fatigue. Increased hair cortisol in sarcoidosis patients was associated with increased psychological distress, assessed by questionnaires. In male sarcoidosis patients, no significant correlations were found between hair testosterone and scores of psychological distress and fatigue. This study suggests that scalp hair cortisol analysis is a feasible, non-invasive biomarker for psychological distress, but not fatigue, in patients with sarcoidosis.

## Supporting information

S1 Dataset Sarcoidosis population. (SAV)

S2 Dataset General population (cortisol, cortisone). (SAV)

S3 Dataset General population (testosterone). (SAV)

S1 File Pearson Correlations Fig 4. (PDF)

S2 File TREND checklist supplementary material. (PDF)

S3 File Protocol version 1 april 2014. (PDF)

## References

[1] Statement on sarcoidosis. Joint Statement of the American Thoracic Society (ATS), the European Respiratory Society (ERS) and the World Association of Sarcoidosis and Other Granulomatous Disorders (WASOG) adopted by the ATS Board of Directors and by the ERS Executive Committee, February 1999. Am J Respir Crit Care Med. 1999;160(2):736–55. Epub 1999/08/03. 10.1164/ajrccm.160.2.ats4-99 . 10430755

[2] Fleischer M, Hinz A, Brahler E, Wirtz H, Bosse-Henck A. Factors associated with fatigue in sarcoidosis. Respir Care. 2014;59(7):1086–94. Epub 2013/11/21. respcare.02080 [pii] 10.4187/respcare.02080 . 24255156

[3] Drent M, Lower EE, De Vries J. Sarcoidosis-associated fatigue. European Respiratory Journal. 2012;40(1):255–63. Epub 2012/03/24. 09031936.00002512 [pii] 10.1183/09031936.00002512 . 22441750

[4] Wilsher ML. Psychological stress in sarcoidosis. Curr Opin Pulm Med. 2012;18(5):524–7. Epub 2012/05/24. 10.1097/MCP.0b013e3283547092 . 22617812

[5] De Vries J, Drent M. Relationship between perceived stress and sarcoidosis in a Dutch patient population. Sarcoidosis Vasc Diffuse Lung Dis. 2004;21(1):57–63. Epub 2004/05/07. . 15127976

[6] Korenromp IH, Heijnen CJ, Vogels OJ, van den Bosch JM, Grutters JC. Characterization of chronic fatigue in patients with sarcoidosis in clinical remission. Chest. 2011;140(2):441–7. Epub 2011/02/19. chest.10-2629 [pii] 10.1378/chest.10-2629 . 21330380

[7] Tsigos C, Chrousos GP. Hypothalamic-pituitary-adrenal axis, neuroendocrine factors and stress. J Psychosom Res. 2002;53(4):865–71. Epub 2002/10/16. S0022399902004294 [pii]. . 12377295 10.1016/s0022-3999(02)00429-4

[8] Cohen S, Kamarck T, Mermelstein R. A global measure of perceived stress. J Health Soc Behav. 1983;24(4):385–96. Epub 1983/12/01. . 6668417

[9] Russell E, Koren G, Rieder M, Van Uum S. Hair cortisol as a biological marker of chronic stress: current status, future directions and unanswered questions. Psychoneuroendocrinology. 2012;37(5):589–601. Epub 2011/10/07. S0306-4530(11)00279-4 [pii] 10.1016/j.psyneuen.2011.09.009 . 21974976

[10] Staufenbiel SM, Penninx BW, Spijker AT, Elzinga BM, van Rossum EF. Hair cortisol, stress exposure, and mental health in humans: a systematic review. Psychoneuroendocrinology. 2013;38(8):1220–35. Epub 2012/12/21. S0306-4530(12)00402-7 [pii] 10.1016/j.psyneuen.2012.11.015 . 23253896

[11] Lasselin J, Laye S, Dexpert S, Aubert A, Gonzalez C, Gin H, et al Fatigue symptoms relate to systemic inflammation in patients with type 2 diabetes. Brain Behav Immun. 2012;26(8):1211–9. Epub 2012/04/04. S0889-1591(12)00067-0 [pii] 10.1016/j.bbi.2012.03.003 . 22469909

[12] DeSantis AS, DiezRoux AV, Hajat A, Aiello AE, Golden SH, Jenny NS, et al Associations of salivary cortisol levels with inflammatory markers: the Multi-Ethnic Study of Atherosclerosis. Psychoneuroendocrinology. 2012;37(7):1009–18. Epub 2011/12/20. S0306-4530(11)00339-8 [pii] 10.1016/j.psyneuen.2011.11.009 22178583 PMC3358540

[13] Korenromp IH, Grutters JC, van den Bosch JM, Heijnen CJ. Post-inflammatory fatigue in sarcoidosis: personality profiles, psychological symptoms and stress hormones. J Psychosom Res. 2012;72(2):97–102. Epub 2012/01/28. S0022-3999(11)00250-9 [pii] 10.1016/j.jpsychores.2011.10.001 . 22281449

[14] Nijhof SL, Rutten JM, Uiterwaal CS, Bleijenberg G, Kimpen JL, Putte EM. The role of hypocortisolism in chronic fatigue syndrome. Psychoneuroendocrinology. 2014;42:199–206. Epub 2014/03/19. S0306-4530(14)00042-0 [pii] 10.1016/j.psyneuen.2014.01.017 . 24636516

[15] Atlantis E, Fahey P, Cochrane B, Wittert G, Smith S. Endogenous testosterone level and testosterone supplementation therapy in chronic obstructive pulmonary disease (COPD): a systematic review and meta-analysis. BMJ Open. 2013;3(8). Epub 2013/08/15. bmjopen-2013-003127 [pii] 10.1136/bmjopen-2013-003127 23943774 PMC3740247

[16] Bercea RM, Mihaescu T, Cojocaru C, Bjorvatn B. Fatigue and serum testosterone in obstructive sleep apnea patients. Clin Respir J. 2015;9(3):342–9. Epub 2014/04/15. 10.1111/crj.12150 . 24725752

[17] Dev R, Hui D, Del Fabbro E, Delgado-Guay MO, Sobti N, Dalal S, et al Association among hypogonadism, symptom burden, and survival in male patients with advanced cancer. Cancer. 2014;120(10):1586–93. Epub 2014/03/01. 10.1002/cncr.28619 24577665 PMC4046338

[18] Spruit MA, Thomeer MJ, Gosselink R, Wuyts WA, Van Herck E, Bouillon R, et al Hypogonadism in male outpatients with sarcoidosis. Respir Med. 2007;101(12):2502–10. Epub 2007/09/15. S0954-6111(07)00322-8 [pii] 10.1016/j.rmed.2007.07.009 . 17855065

[19] Gupta SK, Lindemulder EA, Sathyan G. Modeling of circadian testosterone in healthy men and hypogonadal men. J Clin Pharmacol. 2000;40(7):731–8. Epub 2000/07/07. . 10883414 10.1177/00912700022009486

[20] Weckesser LJ, Plessow F, Pilhatsch M, Muehlhan M, Kirschbaum C, Miller R. Do venepuncture procedures induce cortisol responses? A review, study, and synthesis for stress research. Psychoneuroendocrinology. 2014;46:88–99. Epub 2014/06/03. S0306-4530(14)00144-9 [pii] 10.1016/j.psyneuen.2014.04.012 . 24882161

[21] Wester VL, van Rossum EF. Clinical applications of cortisol measurements in hair. Eur J Endocrinol. 2015;173(4):M1–10. Epub 2015/05/01. EJE-15-0313 [pii] 10.1530/EJE-15-0313 . 25924811

[22] De Vries J, Michielsen H, Van Heck GL, Drent M. Measuring fatigue in sarcoidosis: the Fatigue Assessment Scale (FAS). Br J Health Psychol. 2004;9(Pt 3):279–91. Epub 2004/08/07. 10.1348/1359107041557048 . 15296678

[23] Scholtens S, Smidt N, Swertz MA, Bakker SJ, Dotinga A, Vonk JM, et al Cohort Profile: LifeLines, a three-generation cohort study and biobank. Int J Epidemiol. 2015;44(4):1172–80. Epub 2014/12/17. dyu229 [pii] 10.1093/ije/dyu229 . 25502107

[24] Wester VL, Noppe G, Savas M, van den Akker EL, de Rijke YB, van Rossum EF. Hair analysis reveals subtle HPA axis suppression associated with use of local corticosteroids: The Lifelines cohort study. Psychoneuroendocrinology. 2017;80:1–6. Epub 2017/03/14. S0306-4530(16)30823-X [pii] 10.1016/j.psyneuen.2017.02.024 . 28288364

[25] Noppe G, de Rijke YB, Dorst K, van den Akker EL, van Rossum EF. LC-MS/MS-based method for long-term steroid profiling in human scalp hair. Clin Endocrinol (Oxf). 2015;83(2):162–6. Epub 2015/04/01. 10.1111/cen.12781 . 25823708

[26] de Kleijn WP, De Vries J, Wijnen PA, Drent M. Minimal (clinically) important differences for the Fatigue Assessment Scale in sarcoidosis. Respir Med. 2011;105(9):1388–95. Epub 2011/06/28. S0954-6111(11)00167-3 [pii] 10.1016/j.rmed.2011.05.004 . 21700440

[27] De Vries J, Rothkrantz-Kos S, van Dieijen-Visser MP, Drent M. The relationship between fatigue and clinical parameters in pulmonary sarcoidosis. Sarcoidosis Vasc Diffuse Lung Dis. 2004;21(2):127–36. Epub 2004/07/30. . 15281434

[28] Ware JE Jr., Sherbourne CD. The MOS 36-item short-form health survey (SF-36). I. Conceptual framework and item selection. Med Care. 1992;30(6):473–83. Epub 1992/06/11. . 1593914

[29] Patel AS, Siegert RJ, Creamer D, Larkin G, Maher TM, Renzoni EA, et al The development and validation of the King's Sarcoidosis Questionnaire for the assessment of health status. Thorax. 2013;68(1):57–65. 10.1136/thoraxjnl-2012-201962 . 23065052

[30] Van Manen MJ, Wapenaar M, Strookappe B, Drent M, Elfferich M, de Vries J, et al Validation of the King's Sarcoidosis Questionnaire (KSQ) in a Dutch sarcoidosis population. Sarcoidosis Vasc Diffuse Lung Dis. 2016;33(1):75–82. Epub 2016/04/09. . 27055839

[31] Zigmond AS, Snaith RP. The hospital anxiety and depression scale. Acta Psychiatr Scand. 1983;67(6):361–70. Epub 1983/06/01. . 6880820 10.1111/j.1600-0447.1983.tb09716.x

[32] Short SJ, Stalder T, Marceau K, Entringer S, Moog NK, Shirtcliff EA, et al Correspondence between hair cortisol concentrations and 30-day integrated daily salivary and weekly urinary cortisol measures. Psychoneuroendocrinology. 2016;71:12–8. Epub 2016/05/29. S0306-4530(16)30136-6 [pii] 10.1016/j.psyneuen.2016.05.007 27235635 PMC4955743

[33] Abell JG, Stalder T, Ferrie JE, Shipley MJ, Kirschbaum C, Kivimaki M, et al Assessing cortisol from hair samples in a large observational cohort: The Whitehall II study. Psychoneuroendocrinology. 2016;73:148–56. Epub 2016/08/09. S0306-4530(16)30461-9 [pii] 10.1016/j.psyneuen.2016.07.214 27498290 PMC5052124

[34] Van Uum SH, Sauve B, Fraser LA, Morley-Forster P, Paul TL, Koren G. Elevated content of cortisol in hair of patients with severe chronic pain: a novel biomarker for stress. Stress. 2008;11(6):483–8. Epub 2008/07/09. 794221266 [pii] 10.1080/10253890801887388 . 18609301

[35] Stalder T, Steudte-Schmiedgen S, Alexander N, Klucken T, Vater A, Wichmann S, et al Stress-related and basic determinants of hair cortisol in humans: A meta-analysis. Psychoneuroendocrinology. 2017;77:261–74. 10.1016/j.psyneuen.2016.12.017 . 28135674

[36] Sauve B, Koren G, Walsh G, Tokmakejian S, Van Uum SH. Measurement of cortisol in human hair as a biomarker of systemic exposure. Clin Invest Med. 2007;30(5):E183–91. Epub 2007/09/26. . 17892760 10.25011/cim.v30i5.2894

[37] Wells S, Tremblay PF, Flynn A, Russell E, Kennedy J, Rehm J, et al Associations of hair cortisol concentration with self-reported measures of stress and mental health-related factors in a pooled database of diverse community samples. Stress. 2014;17(4):334–42. Epub 2014/06/07. 10.3109/10253890.2014.930432 . 24903269

[38] Santos A, Crespo I, Aulinas A, Resmini E, Valassi E, Webb SM. Quality of life in Cushing's syndrome. Pituitary. 2015;18(2):195–200. Epub 2015/02/04. 10.1007/s11102-015-0640-y . 25647329

[39] Judd LL, Schettler PJ, Brown ES, Wolkowitz OM, Sternberg EM, Bender BG, et al Adverse consequences of glucocorticoid medication: psychological, cognitive, and behavioral effects. Am J Psychiatry. 2014;171(10):1045–51. Epub 2014/10/02. 1911273 [pii] 10.1176/appi.ajp.2014.13091264 . 25272344

[40] Strookappe B, Swigris J, De Vries J, Elfferich M, Knevel T, Drent M. Benefits of Physical Training in Sarcoidosis. Lung. 2015;193(5):701–8. Epub 2015/08/20. 10.1007/s00408-015-9784-9 [pii]. . 26286208

[41] van Manen MJG, van 't Spijker A, Tak NC, Baars CT, Jongenotter SM, van Roon LR, et al Patient and partner empowerment programme for idiopathic pulmonary fibrosis. European Respiratory Journal. 2017;49(4). 10.1183/13993003.01596-2016 28446554

[42] Sgalla G, Cerri S, Ferrari R, Ricchieri MP, Poletti S, Ori M, et al Mindfulness-based stress reduction in patients with interstitial lung diseases: a pilot, single-centre observational study on safety and efficacy. BMJ Open Respir Res. 2015;2(1):e000065 10.1136/bmjresp-2014-000065 25806113 PMC4360722

[43] Lingner H, Buhr-Schinner H, Hummel S, van der Meyden J, Grosshennig A, Nowik D, et al Short-Term Effects of a Multimodal 3-Week Inpatient Pulmonary Rehabilitation Programme for Patients with Sarcoidosis: The ProKaSaRe Study. Respiration. 2018;95(5):343–53. 10.1159/000486964 . 29486478

[44] Liu D, Ahmet A, Ward L, Krishnamoorthy P, Mandelcorn ED, Leigh R, et al A practical guide to the monitoring and management of the complications of systemic corticosteroid therapy. Allergy Asthma Clin Immunol. 2013;9(1):30 Epub 2013/08/21. 1710-1492-9-30 [pii] 10.1186/1710-1492-9-30 23947590 PMC3765115

