# Supplementary material for: Scalp hair cortisol and testosterone levels in patients with sarcoidosis
Source: PLoS One. 2019 Jun 14;14(6):e0215763. doi: 10.1371/journal.pone.0215763 (PMC6568399; doi:10.1371/journal.pone.0215763)
Supplement: S1 File — (PDF) [file pone.0215763.s004.pdf]

S1 Fig 4. Pearson Correlations

		Cortisol	Cortisone	Testosteron	FAS	PSS	HADS Anxiety	HADS Depression	EQ5D5L	SF-36 MH	SF-36 GH	KSQ GHS	FVC	TLCOc
**Cortisol**	Pearson Correlation		.715**	.381	.137	.306	.466**	.458**	-.260	-.379*	-.275	-.246	-,163	,183
	Sig. (2-tailed)		.000	.199	.454	.094	.010	.010	.151	.032	.127	.174	,459	,514
	N		32	13	32	31	30	31	32	32	32	32	23	15
**Cortisone**	Pearson Correlation	.715**		.259	-.014	-.001	.135	.183	.026	-.050	-.177	-.037	-,353	,303
	Sig. (2-tailed)	.000		.392	.938	.997	.477	.323	.888	.786	.333	.841	,099	,272
	N	32		13	32	31	30	31	32	32	32	32	23	15
**Testosteron**	Pearson Correlation	.381	.259		-.430	-.530	-.353	-.434	.327	.328	-.551	.421	-,756*	-,611
	Sig. (2-tailed)	.199	.392		.143	.076	.260	.158	.276	.274	.051	.151	,018	,198
	N	13	13		13	12	12	12	13	13	13	13	9	6
**FAS**	Pearson Correlation	.137	-.014	-.430		.694**	.635**	.768**	-.642**	-.326	.271	-.822**	,019	-,168
	Sig. (2-tailed)	.454	.938	.143		.000	.000	.000	.000	.069	.134	.000	,931	,550
	N	32	32	13		31	30	31	32	32	32	32	23	15
**PSS**	Pearson Correlation	.306	-.001	-.530	.694**		.911**	.873**	-.639**	-.561**	.102	-.762**	-,024	-,297
	Sig. (2-tailed)	.094	.997	.076	.000		.000	.000	.000	.001	.584	.000	,913	,282
	N	31	31	12	31		29	30	31	31	31	31	23	15
**HADS Anxiety**	Pearson Correlation	.466**	.135	-.353	.635**	.911**		.881**	-.641**	-.694**	-.021	-.817**	,040	-,163
	Sig. (2-tailed)	.010	.477	.260	.000	.000		.000	.000	.000	.911	.000	,857	,561
	N	30	30	12	30	29		30	30	30	30	30	23	15
**HADS Depression**	Pearson Correlation	.458**	.183	-.434	.768**	.873**	.881**		-.785**	-.594**	-.030	-.856**	,018	-,026
	Sig. (2-tailed)	.010	.323	.158	.000	.000	.000		.000	.000	.874	.000	,936	,926
	N	31	31	12	31	30	30		31	31	31	31	23	15
**EQ5D5L**	Pearson Correlation	-.260	.026	.327	-.642**	-.639**	-.641**	-.785**		.407*	.185	.768**	-,283	-,238
	Sig. (2-tailed)	.151	.888	.276	.000	.000	.000	.000		.021	.309	.000	,191	,393
	N	32	32	13	32	31	30	31		32	32	32	23	15
**SF-36 MH**	Pearson Correlation	-.379*	-.050	.328	-.326	-.561**	-.694**	-.594**	.407*		.129	.505**	,021	,171
	Sig. (2-tailed)	.032	.786	.274	.069	.001	.000	.000	.021		.481	.003	,924	,543
	N	32	32	13	32	31	30	31	32		32	32	23	15
**SF-36 GH**	Pearson Correlation	-.275	-.177	-.551	.271	.102	-.021	-.030	.185	.129		-.132	,135	-,196
	Sig. (2-tailed)	.127	.333	.051	.134	.584	.911	.874	.309	.481		.473	,539	,484
	N	32	32	13	32	31	30	31	32	32		32	23	15
**KSQ GHS**	Pearson Correlation	-.246	-.037	.421	-.822**	-.762**	-.817**	-.856**	.768**	.505**	-.132		,021	,171
	Sig. (2-tailed)	.174	.841	.151	.000	.000	.000	.000	.000	.003	.473		,924	,543
	N	32	32	13	32	31	30	31	32	32	32		23	15
**FVC**	Pearson Correlation	-,163	-,353	-,756*	,019	-,024	,040	,018	-,283	,021	,135	-,120		,530*
	Sig. (2-tailed)	,459	,099	,018	,931	,913	,857	,936	,191	,924	,539	,584		,042
	N	23	23	9	23	23	23	23	23	23	23	23		15
**TLCOc**	Pearson Correlation	,183	,303	-,611	-,168	-,297	-,163	-,026	-,238	,021	,135	-,120	,530*	
	Sig. (2-tailed)	,514	,272	,198	,550	,282	,561	,926	,393	,924	,539	,584	,042	
	N	15	15	6	15	15	15	15	15	23	23	23	15	
