# Supplementary material for: Scalp hair cortisol and testosterone levels in patients with sarcoidosis
Source: PLoS One. 2019 Jun 14;14(6):e0215763. doi: 10.1371/journal.pone.0215763 (PMC6568399; doi:10.1371/journal.pone.0215763)
Supplement: S3 File — (PDF) [file pone.0215763.s006.pdf]

RESEARCH PROTOCOL

Hair cortisol and testosterone levels in patients with sarcoidosis associated fatigue

(april 2014)

TABLE OF CONTENTS

1. INTRODUCTION AND RATIONALE 4
2. OBJECTIVES 5
3. STUDY DESIGN 5
4. STUDY POPULATION 5
   - 4.1 Population (base) 5
   - 4.2 Inclusion criteria 5
   - 4.3 Exclusion criteria 6
   - 4.4 Sample size calculation 6
5. METHODS 6
   - 5.1 Study parameters/endpoints 6
     - 5.1.1 Main study parameter/endpoint 6
     - 5.1.2 Secondary study parameters/endpoints (if applicable) 6
   - 5.2 Study procedures 7
   - 5.3 Withdrawal of individual subjects 9
6. STATISTICAL ANALYSIS 9
   - 6.1 Descriptive statistics 9
   - 6.2 Secondary study parameter(s) 9
7. ETHICAL CONSIDERATIONS 9
   - 7.1 Regulation statement 9
   - 7.2 Recruitment and consent 9
   - 7.3 Benefits and risks assessment, group relatedness 10
   - 7.4 Compensation for injury 10
8. ADMINISTRATIVE ASPECTS, MONITORING AND PUBLICATION 10
   - 8.1 Handling and storage of data and documents 10
   - 8.2 Amendments 10
9. INVESTIGATORS 10
10. REFERENCES 11

SUMMARY

Title: Hair cortisol and testosterone levels in patients with sarcoidosis associated fatigue

Rationale: Fatigue is a common symptom in patients with sarcoidosis and has great impact on quality of life (1, 2). Several aspects in the pathogenesis of sarcoidosis and co-morbidities accompanying sarcoidosis could contribute to fatigue. However, until now the etiology of fatigue in sarcoidosis is unknown. Measurements of steroid hormones in scalp hair could possibly be directional in elucidating the underlying mechanism and could also be used as a screening and follow up tool in interventional studies.

Objective: A pilot to investigate whether cortisol and testosterone levels measured in hair differ between patients with sarcoidosis related fatigue, sarcoidosis patients without fatigue and healthy subjects.

Study design: A prospective case-control study, using hair samples of sarcoidosis patients with and without fatigue, compared to an already existing age matched healthy control group.

Study population: Adult sarcoidosis patients at the outpatient clinic of the pulmonary department of the Erasmus MC, Rotterdam

Main study parameters/endpoints: Cortisol and testosterone levels in scalp hair, correlation with Fatigue Assessment Scale.

Nature and extent of the burden and risks associated with participation, benefit and group relatedness: In each participant, a small hair sample will be cut from the posterior vertex of the scalp. Each participant will be asked to fill in five questionnaires. However, this study may contribute in the assessment and management of sarcoidosis related fatigue.

1. INTRODUCTION AND RATIONALE

In patients with sarcoidosis, fatigue is a frequently reported problem (1-3). Fatigue has been reported in 50-70% of the sarcoidosis patients (4). Even when sarcoidosis is clinically in remission, fatigue may remain present and become a chronic problem causing impaired quality of life and reduced socio-economical participation (4, 5). Until now the exact mechanism for chronic fatigue in sarcoidosis remains unknown. Several aspects in the pathogenesis and co-morbidities accompanying sarcoidosis could contribute to the onset of fatigue.

Hypogonadism is one of these possible contributing factors, which is assumed to be present in sarcoidosis (6). Acquired hypogonadism may be caused by systemic inflammation in sarcoidosis, however the underlying process has not yet been elucidated (7, 8). Fatigue and depression have been associated with decreased circulating testosterone levels in male patients with other chronic diseases such as chronic obstructive pulmonary disease (6, 7, 9). Spruit et al. compared the concentration of testosterone in blood serum in male sarcoidosis patients with healthy individuals, but found no differences.(6). However, these blood serum measurements only reflect one moment in time (10) and may therefore not be an adequate marker for long-term testosterone exposure.

Chronic fatigue in sarcoidosis is associated with increased stress levels and as a consequence higher prevalence of psychological symptoms such as depression and anxiety (11, 12). Cortisol is commonly used as a biomarker of stress. Measurement of cortisol levels in serum, urine, saliva or feces is complicated by the fact that these measurements only reflect a moment in time and greatly fluctuate during the day.

Hair analysis is a novel approach of measuring cortisol and testosterone levels. This approach allows a retrospective measurement of the endogenous production of these hormones over longer periods of time, assuming that hair grows at approximately 1 centimeter per month (13-15). Former studies on hair cortisol and chronic stress showed increased hair cortisol levels in many different situations, such as chronic pain or shift work and showed that hair cortisol could serve as a biomarker for chronic stress (13, 16).

To our knowledge there is no experience in using hair biomarkers, such as cortisol and testosterone, in sarcoidosis. This noninvasive method could not only give insight into the mechanism of fatigue in sarcoidosis, but potentially could also be used as a screening and follow up tool in interventional studies.

2. OBJECTIVES

Primary:
A pilot to investigate whether cortisol and testosterone levels measured in hair differ between patients with sarcoidosis related fatigue, sarcoidosis patients without fatigue and healthy subjects.

Secondary:
- To assess if there is there a correlation between hair testosterone and cortisol and scores of different questionnaires as Fatigue Assessment Scale, SF-36, the Kings sarcoidosis questionnaire, EQ-5D-5L and the Perceived Stress Scale.
-To evaluate if hair steroid hormone levels are feasible biomarkers to use in a larger study

3. STUDY DESIGN

This is a prospective observational case-control study at the outpatient clinic of the pulmonary department of the Erasmus MC, Rotterdam. Hair collection and hair cortisol and testosterone measurements will be performed in 10 males and 10 females with sarcoidosis associated fatigue. The control group will consist of 5 males and 5 females with sarcoidosis but without complaints of fatigue. In addition, we will also compare the testosterone- and cortisol in-hair levels with those of an already existing, age matched, control group consisting of healthy age matched adults.

4. STUDY POPULATION

4.1 Population (base)

The study population consists of male and female sarcoidosis patients who meet the inclusion and exclusion criteria outlined below. Patients will be recruited from the outpatient clinic at the Pulmonary Department of the Erasmus MC. Hair steroid values of a previously collected normal values study in adults will be used as healthy controls.

4.2 Inclusion criteria

In order to be eligible to participate in this study, a subject must meet all of the following criteria:

- Age of at least 18 years

- Diagnosis of sarcoidosis based on consistent clinical features/BAL fluid analysis/ PA according to the latest ATS/ERS/WASOG statement on sarcoidosis (17)
- Able to speak, read and write in Dutch
- Presence of sarcoidosis related fatigue, defined as a >=22 score on the Fatigue Assessment Scale (18)
- Other causes of fatigue are excluded or contributing comorbidities are optimally treated (e.g. OSAS, hypothyroidism or anemia)

4.3 Exclusion criteria

A potential subject who meets any of the following criteria will be excluded from participation in this study:

- Unable to understand questionnaires (intellectual impaired or language barrier)
- Hair length < 1 cm
- Use of systemic and/ or inhalation steroids in the last year
- Use of methylphenidate (Ritalin) < 1 month before the study

4.4 Sample size calculation

Not applicable; observational study. All participants who meet the inclusion criteria and provided informed consent for the use of hair samples and clinical data for research purposes will be included in the analysis.

5. METHODS

5.1 Study parameters/endpoints

5.1.1 Main study parameter/endpoint

- Hair cortisol and testosterone levels in sarcoidosis compared to the healthy control group

5.1.2 Secondary study parameters/endpoints (if applicable)

- Fatigue Assessment Scale
- Kings Sarcoidosis Questionnaire
- Perceived Stress Scale
- SF-36

- EQ-5D-5L
- HADS
- Relationship between hair cortisol and clinical parameters such as BMI and waist circumference

5.2 Study procedures

All patients with sarcoidosis and complaints of fatigue with a scheduled visit to the outpatient clinic of the pulmonary department of the Erasmus MC Rotterdam will be informed by the lung physician about the study. If the patient consents to participate he or she will be asked to complete the Fatigue Assessment Scale in order to assess the presence of clinical significant fatigue. Patients fulfilling the inclusion criteria will also be asked to fill out a questionnaire concerning the condition of their hair. Second, a small hair sample will be cut and processed as described below. Third, participants will be asked to fill in the questionnaires mentioned below and return these within two weeks by mail in pre-addressed and stamped envelopes.

<u>Hair processing:</u>

In each subject, a hair sample of approximately 150 hairs is cut from the posterior vertex as close to the scalp as possible using small scissors. Hair samples will be taped on paper and stored in envelopes at room temperature before analysis. Each hair sample will be divided in segments of 1 cm. For each segment, a minimum of 20 mg of hair is weighed and worked up for analysis. After washing each hair sample for 2 min with isopropanol, extraction of steroids will take place in methanol at 26°C for 18 hours. After extraction, the methanol is transferred into clean glass tubes and worked up further using centrifugation and solid phase extraction (SPE). Hair steroid levels will be measured using a Liquid Chromatograpy / Tandem Mass Spectrometry (LC-MS/MS) based method that was recently developed in-house. Steroid levels will be expressed in pg/mg hair

<u>Questionnaires:</u>

Fatigue Assessment Scale

The Fatique Assessment Scale (FAS) is a short questionnaire to assess fatigue. It contains 10 fatigue specific questions that have been validated in patients with sarcoidosis (4, 19). Scores on the FAS can range from 10 to 50, high scores indicating more fatigue. A FAS score of ≥ 22 is considered as significant fatigue (20-22). The MCID is estimated on a change of 4 points indicating that when the FAS

scores of a patient change between two time points with at least 4 points, this change in fatigue is clinically meaningful (19, 23).

EQ-5D-5L:

The EQ-5D-5L is a self-reported standardised generic instrument for health status measurement. It comprises 5 dimensions (mobility, self-care, daily activities, pain and mood) and a Visual Analogue Scale on general health-status. The ratings can be analysed on an individual level using health-state utility scores (24, 25).

SF-36:

The medical outcomes short form 36 (SF 36) is a generic health-related quality of life instrument (26). It consists of 36 items, divided in eight dimensions of quality of life (physical functioning, role physical functioning, role emotional functioning, mental health, vitality, social functioning, bodily pain, and general health). Scale scores range from 0 to 100, a higher score represents better functioning.

Kings Sarcoidosis Questionnaire

This questionnaire is used to assess the impact of sarcoidosis on patients' lives by identifying health status issues seen from the patients perspective (27). The KSQ consists of five modules: General health status (10 items), Lung (6 items), Medication (3 items), skin (3 items) and eye (7 items). The general health status module is always administered, the other items only when applicable. All scores range from 0 to 100, a higher score represents a better health status (27).

Perceived stress scale

The perceived stress scale is the most widely used psychological instrument for measuring the perception of stress. The list contains 14 items. It is a measure of the degree to which situations in one's life are appraised as stressful. (28)

HADS

The Hospital Anxiety and Depression Scale (HADS) comprises a 7-item depression scale and a 7-item anxiety scale. The scores range from 0-21 for either anxiety of depression. The cut-off point of 8/21 is identified for either anxiety or depression (29, 30)

Hair condition questionnaire

Prior to taking hair samples, patients will be asked to fill in a questionnaire about the condition of their hair.

Pulmonary function tests:

The results of routinely measured pulmonary function outcomes (spirometry, carbon monoxide transfer capacity (TLCO), 6-minute-walk-test) will be used when available.

5.3 Withdrawal of individual subjects

At any time patients have the right to withdraw from the study without any repercussion for the ongoing care of the patient. The investigator can decide to withdraw a subject from the study for urgent medical reasons. Patients that drop out will be replaced.

6. STATISTICAL ANALYSIS

6.1 Descriptive statistics

Hair cortisol and testosterone levels will be compared between groups using unpaired student T tests.

6.2 Secondary study parameter(s)

Correlations between hair cortisol and testosterone levels and the FAS, EQ-5D-5L, SF-36, PSS, HADS and Kings Sarcoidosis Questionnaire will be assessed using Pearson's or Spearman's correlation coefficients, where appropriate. Paired and unpaired student T tests will be used to compare the questionnaire outcomes within and between groups.

7. ETHICAL CONSIDERATIONS

7.1 Regulation statement

This study will be performed according to the principles of the Declaration of Helsinki (64th WMA General Assembly, Fortaleza, Brazil, October 2013) and in accordance with the Medical Research Involving Human Subjects Act (WMO).

7.2 Recruitment and consent

Eligible patients with sarcoidosis and complaints of fatigue will be asked if they are interested to participate in this study, and if so, receive verbal and written information about the study. Patients are asked to read the information outside the consultation room

at their own pace. This will allow ample time to consider participation. Written informed consent will be obtained prior to entering the study.

7.3 Benefits and risks assessment, group relatedness

Patients will be asked to donate one hair sample for research purposes and to fill in a set of questionnaires. There will be no risk and the burden is minimal. Participants will not directly benefit from this study. However, this study may contribute in the assessment and management of sarcoidosis related fatigue.

7.4 Compensation for injury

The sponsor/investigator has a liability insurance which is in accordance with article 7, subsection 6 of the WMO.

8. ADMINISTRATIVE ASPECTS, MONITORING AND PUBLICATION

8.1 Handling and storage of data and documents

All participants will receive an identification number, corresponding to the measured data. The identification number is coupled to the individual background information of the patient which is anonymously stored in a separate document, only available to researchers participating in the study. In publications the patient and controls will not be identifiable. The samples containing cortisol extracts from hair will be stored for a maximum of 15 years.

8.2 Amendments

Amendments are changes made to the research after a favourable opinion by the accredited METC has been given. All amendments will be notified to the METC that gave a favourable opinion. Non-substantial amendments will not be notified to the accredited METC and the competent authority, but will be recorded and filed by the sponsor.

9. INVESTIGATORS

Drs. J.W. Kraan, coordinating investigator
Dr. Ir. R.T. van Domburg, clinical epidemiologist, Erasmus MC Rotterdam
Drs. V.L. Wester, co-investigator
Dr. E.F.C. van Rossum, co-investigator
Dr. M.S. Wijsenbeek, pulmonary physician specialised in ILD, Erasmus MC, Rotterdam

10. REFERENCES

1. Fleischer M, Hinz A, Brähler E, Wirtz H, Bosse-Henck A. Factors associated with fatigue in sarcoidosis. Respir Care. 2013 2013/11/19/.
2. Sharma OP. Fatigue and sarcoidosis. Eur Respir J. 1999 1999/04//;13(4):713-4.
3. de Kleijn WP, De Vries J, Lower EE, Elfferich MD, Baughman RP, Drent M. Fatigue in sarcoidosis: a systematic review. Curr Opin Pulm Med. 2009 Sep;15(5):499-506.
4. Drent M, Lower EE, De Vries J. Sarcoidosis-associated fatigue. Eur Respir J. 2012 2012/07//;40(1):255-63.
5. Korenromp IHE, Heijnen CJ, Vogels OJM, van den Bosch JMM, Grutters JC. Characterization of chronic fatigue in patients with sarcoidosis in clinical remission. Chest. 2011 2011/08//;140(2):441-7.
6. Spruit MA, Thomeer MJ, Gosselink R, Wuyts WA, Van Herck E, Bouillon R, et al. Hypogonadism in male outpatients with sarcoidosis. Respir Med. 2007 2007/12//;101(12):2502-10.
7. Van Vliet M, Spruit MA, Verleden G, Kasran A, Van Herck E, Pitta F, et al. Hypogonadism, quadriceps weakness, and exercise intolerance in chronic obstructive pulmonary disease. Am J Respir Crit Care Med. 2005 2005/11/01/;172(9):1105-11.
8. Fraietta R, Zylberstejn DS, Esteves SC. Hypogonadotropic hypogonadism revisited. Clinics (Sao Paulo). 2013 2013;68 Suppl 1:81-8.
9. Wagner GJ, Rabkin JG, Rabkin R. Testosterone as a treatment for fatigue in HIV+ men. Gen Hosp Psychiatry. 1998 Jul;20(4):209-13.
10. Thomson S, Koren G, Van Steen V, Rieder M, Van Uum SH. Testosterone concentrations in hair of hypogonadal men with and without testosterone replacement therapy. Ther Drug Monit. 2009 Dec;31(6):779-82.
11. De Vries J, Drent M. Relationship between perceived stress and sarcoidosis in a Dutch patient population. Sarcoidosis Vasc Diffuse Lung Dis. 2004 Mar;21(1):57-63.
12. Wilsher ML. Psychological stress in sarcoidosis. Curr Opin Pulm Med. 2012 Sep;18(5):524-7.
13. Russell E, Koren G, Rieder M, Van Uum S. Hair cortisol as a biological marker of chronic stress: current status, future directions and unanswered questions. Psychoneuroendocrinology. 2012 2012/05//;37(5):589-601.
14. Meyer JS, Novak MA. Minireview: Hair cortisol: a novel biomarker of hypothalamic-pituitary-adrenocortical activity. Endocrinology. 2012 2012/09//;153(9):4120-7.
15. Sauve B, Koren G, Walsh G, Tokmakejian S, Van Uum SH. Measurement of cortisol in human hair as a biomarker of systemic exposure. Clin Invest Med. 2007;30(5):E183-91.
16. Staufenbiel SM, Penninx BWJH, Spijker AT, Elzinga BM, van Rossum EFC. Hair cortisol, stress exposure, and mental health in humans: a systematic review. Psychoneuroendocrinology. 2013 2013/08//;38(8):1220-35.
17. Statement on sarcoidosis. Joint Statement of the American Thoracic Society (ATS), the European Respiratory Society (ERS) and the World Association of Sarcoidosis and Other Granulomatous Disorders (WASOG) adopted by the ATS Board of Directors and by the ERS Executive Committee, February 1999. Am J Respir Crit Care Med. 1999 Aug;160(2):736-55.
18. De Vries J, Michielsen H, Van Heck GL, Drent M. Measuring fatigue in sarcoidosis: the Fatigue Assessment Scale (FAS). Br J Health Psychol. 2004 Sep;9(Pt 3):279-91.
19. de Kleijn WP, De Vries J, Wijnen PA, Drent M. Minimal (clinically) important differences for the Fatigue Assessment Scale in sarcoidosis. Respir Med. 2011 Sep;105(9):1388-95.
20. de Kleijn WP, Drent M, De Vries J. Nature of fatigue moderates depressive symptoms and anxiety in sarcoidosis. Br J Health Psychol. 2013 May;18(2):439-52.
21. Michielsen HJ, De Vries J, Van Heck GL. Psychometric qualities of a brief self-rated fatigue measure: The Fatigue Assessment Scale. J Psychosom Res. 2003 Apr;54(4):345-52.

22. De Vries J, Rothkrantz-Kos S, van Dieijen-Visser MP, Drent M. The relationship between fatigue and clinical parameters in pulmonary sarcoidosis. Sarcoidosis Vasc Diffuse Lung Dis. 2004 Jun;21(2):127-36.
23. Jaeschke R, Singer J, Guyatt GH. Measurement of health status. Ascertaining the minimal clinically important difference. Control Clin Trials. 1989 Dec;10(4):407-15.
24. Devlin NJ, Krabbe PF. The development of new research methods for the valuation of EQ-5D-5L. Eur J Health Econ. 2013 Jul;14 Suppl 1:S1-3.
25. Dolan P. Modeling valuations for EuroQol health states. Med Care. 1997 Nov;35(11):1095-108.
26. Ware JE, Jr., Sherbourne CD. The MOS 36-item short-form health survey (SF-36). I. Conceptual framework and item selection. Med Care. 1992 Jun;30(6):473-83.
27. Patel AS, Siegert RJ, Creamer D, Larkin G, Maher TM, Renzoni EA, et al. The development and validation of the King's Sarcoidosis Questionnaire for the assessment of health status. Thorax. 2013 Jan;68(1):57-65.
28. Cohen S, Kamarck T, Mermelstein R. A global measure of perceived stress. J Health Soc Behav. 1983 Dec;24(4):385-96.
29. Bjelland I, Dahl AA, Haug TT, Neckelmann D. The validity of the Hospital Anxiety and Depression Scale. An updated literature review. J Psychosom Res. 2002 Feb;52(2):69-77.
30. Snaith RP, Zigmond AS. The hospital anxiety and depression scale. Br Med J (Clin Res Ed). 1986 Feb 1;292(6516):344.
